# Utilitarianism and the ethical foundations of cost-effectiveness analysis in resource allocation for global health

**DOI:** 10.1186/s13010-019-0074-7

**Published:** 2019-04-03

**Authors:** Elliot Marseille, James G. Kahn

**Affiliations:** 1 Health Strategies International, Oakland, CA USA; 2 Philip R. Lee Institute for Health Policy Studies, University of California, San Francisco, CA USA

**Keywords:** Ethics, Global health, Cost-effectiveness, Utilitarianism, Health economics

## Abstract

Efficiency as quantified and promoted by cost-effectiveness analysis sometimes conflicts with equity and other ethical values, such as the “rule of rescue” or rights-based ethical values. We describe the utilitarian foundations of cost-effectiveness analysis and compare it with alternative ethical principles. We find that while fallible, utilitarianism is usually superior to the alternatives. This is primarily because efficiency – the maximization of health benefits under a budget constraint – is itself an important ethical value. Other ethical frames may be irrelevant, incompatible with each other, or have unacceptable implications. When alternatives to efficiency are considered for precedence, we propose that it is critical to quantify the trade-offs, in particular, the lost health benefits associated with divergence from strict efficiency criteria. Using an example from HIV prevention in a high-prevalence African country, we show that favoring a rights-based decision could result in 92–118 added HIV infections per $100,000 of spending, compared to one based on cost-effectiveness.

## Background

Economic efficiency is a leading criterion for resource allocation decisions for global (or public) health [1, 2]. Yet assessments of efficiency in the form of cost-effectiveness (CEA) or cost-benefit analysis (CBA) are regarded with a mixture of enthusiasm and suspicion: enthusiasm, because, all else equal, program managers and policy makers seek to maximize the benefit from limited dollars; and suspicion, because all else is rarely equal, when other considerations are included such as fairness and reduction in disparities. Indeed, CEAs are perceived by some as ethically suspect because they rest on a conceptual foundation that violates everyday moral intuitions [3]. By representing human life in dollar terms and choosing among life-saving interventions based on mathematically-derived return-on-investment metrics, they undermine the expression of communitarian values, and often appear to conflict with a range of ethical principles including equity, urgent need, and human rights as enshrined in international law [4, 5].

In this article, we describe the ethical framework implied by CEA, utilitarianism, as applied to global health. Second, after describing some of the moral objections to utilitarianism, we show that the criticisms leveled against CEA on ethical grounds are often misleading: Efficiency in promoting human life and health is itself a valid ethical standard, and that alternative formal ethical approaches, as well as everyday ethical intuitions, present their own problems when applied to real world situations. In particular, we discuss the moral equivalence between identified and statistical lives implied by utilitarianism. Using the example of the female condom to prevent HIV transmission in developing countries, we also show that using non-efficiency based principles to guide resource allocation, even central principles such as human rights, when applied too narrowly, can inadvertently lead to undermining those very principles. Third, we argue that while no infallible theory of the ethics of resource allocation is yet available, the utilitarian framework underlying CEA and CBA generally provides trustworthy guidance; is usually superior to the alternatives, and should therefore constitute the default perspective. Finally, we propose that when utilitarianism generates results that stakeholders deem ethically unacceptable, the grounds for that dissatisfaction should be made numerically explicit. A good faith effort should be made to describe and quantify the trade-offs associated with decisions that diverge from efficiency criteria.

### Ethical framework implied by cost-effectiveness analysis

The ethical foundation of CEA and CBA, utilitarianism, was originally developed by the nineteenth century British philosophers Jeremy Bentham and John Stuart Mills [6, 7] and was recently revisited and advanced by Peter Singer in “Practical Ethics” and other writings, and by Joshua Greene in “Moral Tribes” [8, 9].

Utilitarianism is a species of Consequentialism, which is the ethical doctrine that one should judge actions by the outcomes that can reasonably be expected to follow, and not by the actors’ intention or by fidelity to an abstract moral principle. Thus, the overarching maxim of Utilitarianism is, “Act in such a way as to generate the maximum quantum of well-being, happiness, or utility”, or in Bentham’s famous dictum, “the greatest good for the greatest number” [6]. In the context of global health, this implies:

1. Resources should be allocated consistent with maximizing overall benefit, such as deaths averted or quality-adjusted life-years gained. Such allocation decisions are consistent with the findings of cost-effectiveness analyses.
2. All lives have the same value. While anodyne at first sight, this concept helps fuel utilitarianism’s reputation for replacing common humanity with hyper-rational calculation. It means, for example, that there is no basis for distinguishing between identified lives (e.g., a sick person treated at a hospital) versus statistical lives (e.g., unknown individuals who avoid disease). We argue that in general, this principle is in fact consistent with our humanity and is at the heart of the unique contribution of the global health perspective.
3. No special claim accrues to alleviating inequality. The exception is when privileging vulnerable populations, or those with less access to care, is an efficient means to achieving #1, above. This seeming indifference to the plight of the poor may be part of the reason for the “.. . widespread suspicion that CEA does favor the healthy and well-to-do” [10]. We will argue that there is less to object to here than meets the eye: in most populations, efficiency and inequality alleviation are concordant. This is because poor people are usually sicker and start with fewer health resources than wealthier neighbors.

Few would dispute the notion that #1 is an important part of rational resource allocation. Our difference with some critics of CEA is that we believe other ethical values should onely rarely substantially modify guidance based on efficiency alone. As expounded below, those other ethical values are not necessarily of a higher order than efficiency, and have their own problems.

Regarding #2, powerful, often irresistible emotions, especially empathy, impel decision makers to privilege identified lives. Nevertheless, we are aware of no rational basis for elevating this sense of empathy to a principle that should guide policy. Public health as distinct from clinical medicine is, at its core, concerned with populations, not identified individuals. Statistical lives are in fact identified lives for the friends and family members of those persons. The impulse to favor identified individuals is thus a failure of imagination. For public health professionals to treat identified lives as having a primary claim because they are less visible to those professionals, lacks ethical foundation.

Regarding #3, as a practical matter, the area of potential conflict between equity and utilitarianism is smaller than one might expect. This is because the incremental benefit of investing additional global health dollars for the poor and others with limited access to health care is generally greater than the incremental benefits generated by the same dollars spent on the more affluent and those with better access to health care [2]. Thus, in most cases, efficiency and equity goals are aligned. For example, malaria and neglected tropical diseases account for 15% of the total burden of disease in sub-Saharan Africa [11] and malaria deaths are closely associated with poverty [12]. A 2013 systematic review and meta-analysis found a roughly two-fold higher risk of parasitemia in children of lower compared with children of higher socio-economic status [13]. Malaria treatment and prevention is one of the areas where investment in global health have seen the greatest returns in the past 20 years. These interventions are included in the “enhanced investment scenario” for low-income countries needed to achieve global health convergence by 2035 [12].

### Limitations to criticisms of cost-effectiveness analysis and problems with other ethical principles

Various ethical principles are cited to justify policy positions or resource allocation decisions in global health [5]. Each represents a broadly shared moral intuition. However, the policy choices implied by these different principles often conflict. Current public health discourse makes no reference to a meta-ethic which might lead us to choose one ethical principle over another, and thus invites an ad hoc application of decision rules. Policy actors and advocates often choose whatever ethical principle aligns with their existing action preferences. Rather than ethical argument fulfilling its proper function of constraining and informing political and ideological predispositions, the invocation of ethical principles can devolve into justification of those predispositions. At their worst, they may be used to deprecate opponents into silence. For example, because rights are associated with non-negotiable moral and legal imperatives, framing a choice in human rights terms can appear to preclude inquiry and analysis.

A 2012 article by Ruth Macklin and Ethan Cowan outlined several ethical principles that could be brought to bear on the question of how, in developing countries, limited supplies of antiretroviral drugs should be divided between HIV/AIDS treatment and prevention via pre-exposure prophylaxis [14]. The piece concisely describes six ethical principles: utilitarianism, equity, urgent need, prioritarianism, rule of rescue, and equal worth to which we have added a seventh, “Rights”.

1. Utilitarianism: Maximize total health benefit given the relevant budget.
2. Equity: Divide resources to reduce disparities in health status among different groups or strata, poor, women, rural areas, ethnic/racial minorities.
3. Urgent need: More urgent needs give rise to stronger moral claims
4. Prioritarianism: Provide resources to the least advantaged.
5. Rule of rescue: Identified lives in imminent danger take precedence.
6. Equal worth: All lives have equal worth; therefore, all are entitled to the same resources.
7. Rights: Certain freedoms and protections under the law, or material goods and services are due all human beings.

**Fig. 1:**
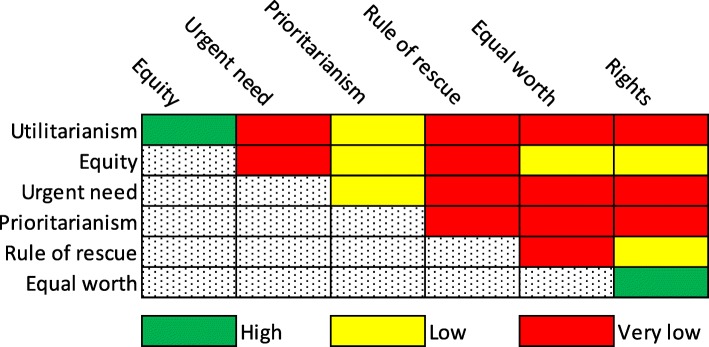
Likelihood of convergence between ethical principles

The authors then discuss the difficulties in interpreting each of these in the context of this HIV/AIDS-related policy decision. For example, if one were to adopt the principle of “urgent need” it is unclear whose needs (those benefiting from treatment versus from prevention) should be considered most urgent. Similarly, it is hard to know what is “equitable” since we have no guidance for choosing equal inputs over equal health effects, or vice versa. Furthermore, does “equity” mean equality, or does it refer to justice based on another unstated criterion? Likewise, too much weight applied to the rule of rescue would quickly exhaust available funds, and thus seriously undermine the ability to build sustainable health systems, and thus progress towards the utilitarian goal of maximizing health, or right-based access to health care. Similar questions and objections apply to each of the principles outlined. It is worth noting that no mention is made by Macklin and Cowan of human rights. But the practical meaning of “rights” in this context would also be difficult to interpret. Does the right to treatment supersede the right to prevention? If so, why? If there is an ethical imperative to provide both adequate treatment and prevention, we’ve assumed away the trade-off, which is at the heart of this and of all resource allocation decisions See Fig. 1.

The figure above shows in rough, qualitative fashion how well these principles coincide in the case of the allocation of antiretroviral medications for HIV prevention via Pre-Exposure Prophylaxis (PrEP) versus HIV treatment. These principles have different possible practical interpretations, particularly Equity, Urgent Need, and Prioritarianism. While the article offers a clear statement of the problem (i.e., the difficulty in choosing among ethical theories and in understanding how each should be interpreted in the context of a specific program choice), the arguments advanced for the selected option are unconvincing. The authors, citing Macklin’s own earlier work write,

“When principles conflict, it becomes necessary to balance competing concerns. There is no correct way of achieving this balance. Moreover, there is no consensus on how the different principles ought to be weighted, or on what weight should be given to the goal of maximizing health compared to other social goods such as education or environmental protection.”

This problem has also been identified by other analysts such as Johri and Norheim in their systematic review of efforts to integrate concerns for equity with cost-effectiveness analysis [15]. They point out that, “A central problem relates to the fact that equity is understood in multiple ways, each demarcating a distinct set of intuitions concerning fairness. Each method takes a distinct approach to how values should articulate with cost-effectiveness evidence.”

Macklin and Cowan advocate for balancing the influence of multiple principles. Akin to advancing an unfalsifiable hypothesis, the appeal to weighing moral desiderata based on unspecified criteria makes it hard to know under what circumstances a choice can be said to be ethically incorrect. Later we find, “The ultimate goal, of course, is to achieve a utilitarian outcome tempered by considerations of equity and urgent need.” Why “of course”? If this conclusion flows naturally from ordinary moral intuitions, why attempt to identify and apply general principles? The proposed conclusion is only a re-statement of the original problem, begging the question, “In what proportions should these principles be mixed, and what principle would guide us to the optimal proportions?” The conclusion advocated in the article, namely that treatment has a prior claim over prevention in the allocation of antiretroviral drugs for HIV/AIDS, is simply asserted based on an opaque weighting of competing ethical principles. The unstated meta-ethical principle is that a result that highly over-weights any one principle is likely to be unethical. But why should that be? Why is it not just as plausible that one principle should dominate? Since this question is not addressed, we are left with the ad hoc mix of ethical appeals that policy makers now use. In the absence of guidance on how to weight the multiple ethical principles described, decisions based on historical and cultural norms, politics and ideology can be dressed in whatever ethical clothing seems to fit. This is a description of the world we now inhabit. It is unclear how “balancing” multiple competing ethical principles puts health resource allocation on a more reliable ethical foundation.

A set of thought experiments dubbed the “Trolley Problem” was advanced by the British philosopher Philippa Foot at Oxford University in 1967 and later elaborated by Judith Thomson at MIT and others [16–19]. The trolley problems demonstrate vividly that different respondents consider different circumstances to be morally relevant. More tellingly, the same respondent may find acceptable a principle such as “one must take no action that results in the death of an innocent” in one set of circumstances, only to be repulsed by the consequences of applying such a principle under slightly altered circumstances.^1^ If contemplation of “Trolleyology” reduces one’s confidence in everyday ethical judgments, Joshua Greene, in *Moral Tribes*, [9] and Randall Moore in his 1996 article, *Caring for Identified* Versus *Statistical Lives: An Evolutionary View of Medical Distributive Justice* [20] advance the idea that far from being a direct line to sound ethical understanding, ordinary ethical intuitions are a function of evolutionary pressures of the ancient savannah, especially the need to interact successfully with small numbers of fellow tribe members.

Humans, including scientists trained in statistics, also handle small numbers much better than large numbers (Tversky and Kahneman 1971). This is consistent with our evolutionary need to deal with small numbers of people and objects and not with large numbers. Throughout the great majority of human history, an ability to easily understand and manipulate large numbers would have been wasted and wasteful. We would expect that evolution did not emphasize cognitive abilities that would be little used, because such an emphasis would waste neurological energy that could be spent on more practical tasks [20].

The theory that different types of cognitive processes are used to make judgments about specific as opposed to general objects is supported by a number of psychological theories, [21, 22] and dual-process models [23, 24]. A discussion of the state of the neuroscience relevant to these claims is beyond the scope of this article. However, on this account, the general tendency to accord preferential treatment to identified lives [25, 26] may be a function of the evolution of “moral brains” molded by the survival advantage conferred by forming strong affiliations and reciprocal loyalties, not on recognizable principles of justice.

The single most common objection to utilitarianism is that it seems to permit using human beings as a means to an end, thus violating the rights of those people [9]. John Rawls, in particular, criticized utilitarianism on these grounds [27]. For example, if it could be shown that the unhappiness of slaves was outweighed by the happiness of slave owners, would slavery therefore be morally justified? This is the kind of counter-intuitive, and indeed, repugnant outcome that utilitarian rationality sometimes appears to imply. On the other hand, utilitarianism can also seem to place demands on moral actors that appear too stringent. The logical terminus of Peter Singer’s famous thought experiment regarding what we should be willing to give up to save a drowning child, is that we should be willing to impoverish ourselves in order to save the lives of the poor and sick in less developed countries [28]. These and other arguments against utilitarianism and replies to these objections are summarized in *Moral Tribes.* For example, according to Greene, slavery is actually not permitted on the utilitarian account because, as a practical matter, it is implausible that slavery could increase net utility, though it might increase wealth.

### Arguments for utilitarianism as the default ethical perspective

The possibility of repugnant outcomes is by no means unique to utilitarianism. As shown in the Trolley Problem, and in evaluating the ethical implications of favoring expenditures on antiretroviral drugs for treatment over pre-exposure prophylaxis, non-efficiency based principles are often hard to interpret, irrelevant, or contradictory. They may also lead to outcomes that diverge dramatically from that of health-maximization. Cost-effectiveness analysis has the virtue of being relevant to any resource allocation decision. Its operational definition is unambiguous (maximization of health benefits for a given budget) even if performing cost-effectiveness calculations is sometimes challenging.

Other principles, such as rule of rescue or the urgent need based adjudications of claims on health care resources, have expression in clinical medicine and elsewhere. However, the principles appropriate when considering the welfare of large populations differs from those appropriate for clinical medicine or for small communities and families. Utilitarianism, because it does not distinguish between identified and statistical lives is, in general, the framework best suited to the former.

We do not propose that utilitarianism is the only legitimate guide to global health resource allocation decisions. However, we do suggest that it should be the point of departure for further analysis. Because of its intrinsic ethical dimensions, efficiency is not merely one criterion among many. The promotion of human flourishing is a central goal of most ethical system. Attaining the greatest population health available with given resources is consonant with that flourishing. Thus, decisions to diverge from pursuit of that goal to promote other ethical values should be acknowledged and justified.

Wherever possible, decision-makers should quantify the tradeoff, i.e. the loss of health resulting from pursuit of other ethical values. This will often be possible in a rough but serviceable manner. For example, spending incremental dollars on the male condom will almost always generate greater health benefit than spending the same money on female condoms [29]. The details of why this is true in almost every HIV epidemic type and risk sub-population are complex, but this finding was driven primarily by two factors: a) the female condom is much more expensive than the male condom while conferring the same protective benefit per unprotected sex episode; and b) use of a female condom often displaces use of a male condom, thus providing no additional protection. However, one of the virtues of the female condom according to proponents, is that it enhances women’s autonomy in negotiating the terms of sexual relations, and thus contributes to the empowerment and rights of women [30, 31]. Though potentially significant, the degree to which access to the female condom helps secure this right to autonomy is hard to quantify. Rather than either ignoring right-based imperatives on the one hand, or insisting that these values trump “mere” efficiency concerns on the other hand, we performed an analysis which solved for the value that would need to be placed on the incremental empowerment of women, such that investments in the female condom over the male condom would be justified. Table 1 illustrates the health consequences of promoting the female condom over the male condom in high-prevalence HIV countries. The figures are the results of a cost-effectiveness model that assumes the full costs of $0.13 for the male condom and $1.00 for the female condom. This is a low estimate of the cost of the female condom and the results displayed are therefore likely tilted in favor of the female condom. The model incorporates information on HIV transmission risk per episode; protective benefits of both types of condoms; sexual behavioral data on three sub-populations, sex workers, women with regular partners and women with casual partners; rates of substitution between male and female condoms; and other parameters affecting the cost of generating an incremental protected sexual episode.

**Table 1:**
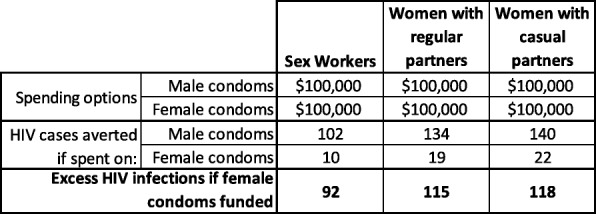
Comparison of the effect of spending 100,000 on the female condom versus spending 100,000 on the male condom in three HIV risk groups in high-prevalence countries

The key finding is that for every $100,000 spent on condoms in an African country with high HIV prevalence, between 92 and 118 additional cases of HIV could be averted by investing in the male condom rather than the female condom. If one believes in the power of the female condom to enhance women’s rights, one might want to argue that this is a price worth paying. It would be an example of what Johri terms “the opportunity cost of equity” [15]. However, in the context of the female condom it seems a difficult case to make, particularly if one is concerned about the rights and health, of those 92–118 additionally infected people, many of whom will be women. Article 25 of the United Nations Universal Declaration of Human Rights guarantees access to adequate medical care for all persons [32]. Similar language exists in Articles 12 and14 of the Convention on the Elimination of All Forms of Discrimination Against Women, and Article 12 of the International Covenant on Economic, Social and Cultural Rights [33, 34]. These legal and moral principles are not superseded by utilitarian values. But the full realization of these rights-based values will, for the foreseeable future be imperfect given health care budgets and other constraints. Therefore, efficiency concerns as expressed in utilitarianism and cost-effectiveness analysis will often be the best guide to rapidly securing those rights for as many people as possible. However, this can only be accomplished when decision makers acknowledge that the trade-offs of the type illustrated in the female condom example are real and consequential.

When competing ethical principles favor different actions, following non-efficiency based principles may increase mortality or morbidity. It is true that a small fraction of what the world spends on armaments and on ultra-luxurious or frivolous pursuits could, if re-deployed, have huge global health benefits. But this information is of no use to the Minister of Health in a low-income country as she decides what portion of her budget should be allocated to TB drugs, versus bed nets to control malaria.

## Conclusions

The long-term social and political project of re-directing resources away from activities that undermine human flourishing and toward those that are conducive, is one of the most urgent of our era. However, for any meaningful time horizon there will be insufficient money to pursue all beneficial activities. Trade-offs, and the problems of resource allocation will therefore persist. Utilitarianism will usually be the most reliable guide in resolving those trade-offs.

## Abbreviations

CBA: Cost-benefit analysis
CEA: Cost-effectiveness analysis
HIV: Human Immunodeficiency Virus
PrEP: Pre-Exposure Prophylaxis

## Footnotes

1 Once the domain of philosophers and ethicists, “Trolleyology” is now widely discussed in the popular press. This may be in part because the types of fanciful situations concocted in the original trolley problems are now similar to actual dilemmas that must be addressed by those writing the software governing autonomous vehicles.
